# The Prognostic Significance of Lymphatics in Colorectal Liver Metastases

**DOI:** 10.1155/2014/954604

**Published:** 2014-05-20

**Authors:** Vijayaragavan Muralidharan, Linh Nguyen, Jonathan Banting, Christopher Christophi

**Affiliations:** Department of Surgery, The University of Melbourne and Austin Hospital, Lance Townsend Building Level 8, Studley Road, Heidelberg, Melbourne, VIC 3084, Australia

## Abstract

*Background*. Colorectal Cancer (CRC) is the most common form of cancer diagnosed in Australia across both genders. Approximately, 40%–60% of patients with CRC develop metastasis, the liver being the most common site. Almost 70% of CRC mortality can be attributed to the development of liver metastasis. This study examines the pattern and density of lymphatics in colorectal liver metastases (CLM) as predictors of survival following hepatic resection for CLM. *Methods*. Patient tissue samples were obtained from the Victorian Cancer Biobank. Immunohistochemistry was used to examine the spatial differences in blood and lymphatic vessel densities between different regions within the tumor (CLM) and surrounding host tissue. Lymphatic vessel density (LVD) was assessed as a potential prognostic marker. *Results*. Patients with low lymphatic vessel density in the tumor centre, tumor periphery, and adjacent normal liver demonstrated a significant disease-free survival advantage compared to patients with high lymphatic vessel density (*P* = 0.01, *P* > 0.01, and *P* = 0.05, resp.). Lymphatic vessel density in the tumor centre and periphery and adjacent normal liver was an accurate predictive marker of disease-free survival (*P* = 0.05). *Conclusion*. Lymphatic vessel density in CLM appears to be an accurate predictor of recurrence and disease-free survival.

## 1. Introduction

Almost 40%–60% of patients with colorectal cancer (CRC) develop metastasis, predominantly in the liver [1]. Tumor angiogenesis has been implicated as a major factor in the development and spread of these metastases. The recent discovery of vascular endothelial growth factor-C (VEGF-C) and VEGF receptor-3 (VEGFR-3) involvement in lymphatic vessel development [2] and specific lymphatic markers has provided new insights into the field of lymphangiogenesis [3]. Using these markers, studies have suggested that lymphangiogenesis plays an active role in the formation and spread of colorectal liver metastases (CLM) [4, 5]. The patterns of intratumoral lymphatics may have potential clinical significance as a predictive marker of disease recurrence and patient survival [5].

In this study, we investigated the patterns of tumor lymphangiogenesis using monoclonal D2-40 and LYVE-1 antibody, as a predictive marker for disease-free survival in patients with CLM. Blood vessels were examined using CD34 antibody to differentiate the blood vessels from the lymphatic vessels.

## 2. Patients and Methods

### 2.1. Case Selection

Tissue was obtained from the Victorian Cancer Biobank for 49 patients who underwent hepatic resection for CLM. Informed consent had been obtained from these patients at the time of surgery for long-term storage of specimen samples and subsequent research according to well-established protocols. Formal ethics approval was obtained from the Human Research Ethics Committee (HREC) of the Austin Hospital for the study (HREC submission number: H2012/04618). Patients' demographic information was obtained from data records.

### 2.2. Definition of Tumor Region

The tumor mass was divided into four regions for each parameter measured; tumor periphery, tumor centre, liver immediately adjacent to tumor, and host liver distal to tumor. Previously, we demonstrated that, following the treatment with a novel vascular destructive agent (VDA), OXi4503, the bulk of the tumor died leaving a viable rim of tumor cells at the periphery extending one hundred microns from the tumor-host interface towards the tumor centre [6]. Based on findings from our previous study, the tumor periphery is defined as the area covering the tumor-host interface and extending one hundred microns towards the tumor centre. The tumor centre is the remaining bulk of the tumor without considering the periphery. The liver immediately adjacent to the tumor is defined as the area at the tumor-host interface and extending one hundred microns away from the tumor. The host liver distal to the tumor is host liver which is farther than one hundred microns from the tumor.

### 2.3. Immunohistochemistry

Tissue samples from the specimen were fixed in 10% buffered formalin, embedded in paraffin, and cut into four *μ*m thick sections. Sections were deparaffinized and rehydrated using standard techniques. Endogenous peroxidases were blocked using 3% peroxide for 30 minutes at room temperature.

D2-40 is a new selective monoclonal antibody for lymphatic endothelium which does not cross react with blood vessel endothelium [7]. For D2-40 and LYVE-1 staining, heat antigen retrieval was performed using TRIS buffer (50 mM) pH 9.5 for 15 minutes at 99°C. The sections were immunostained with mouse monoclonal antibody D2-40 used at 0.03475 mg/mL (D2-40, Dako, Victoria, Australia) and LYVE-1 used at 0.0067 mg/mL (Abcam, Cambridge, USA).

CD34 is an established endothelial vessel marker normally expressed on tumor vessels and host vessels undergoing neovasculature [8]. For CD34, no antigen retrieval was required. Normal goat serum (20%) was used to block nonspecific binding. CD34 was used at 0.005 mg/mL (Abcam, Cambridge, USA).

A polymer-based detection kit containing goat anti-mouse immunoglobulins (IgG) coupled with horseradish peroxidase (HRP) (ENvision Plus, DakoCytomation Pty, Ltd, Botany, NSW, Australia) was used. The presence of vessels was visualized using diaminobenzidine (DAB) as a substrate. Appropriate negative controls were done simultaneously for each batch of slides.

### 2.4. Quantitation

Images of positively stained vessels were captured using a digital light microscope (Nikon Coolscope, Nikon Corporation, Japan) between 10x and 400x magnification. The images of tumor fields were captured to be representative of the entire tumor, using a raster pattern which allowed for captured fields to be random and avoid overlap. Between 20 and 30 fields per tumor were assessed. The images were analyzed using Image-Pro Plus (Version 5, Media Cybernetics, Perth Australia).

Vessels were assessed as the number of positively stained vessels per tumor area to provide a microvascular density index.

The lymphatic vessel density (LVD) assessments were performed by researchers blinded to the patient outcome data.

### 2.5. Statistical Analysis

All data are expressed as the mean ± SEM unless otherwise stated. Data were tested for normality. Pairwise comparisons of group means for parametric data were performed using analysis of variance (ANOVA) with post hoc analysis as appropriate. Nonparametric data were performed using Mann-Whitney* U* or Kruskal-Wallis tests, as appropriate.

Disease-free survival was calculated from the date of surgery to the date of progression or the date of last follow up. Overall survival was not used in this study as a result of a relatively small number of deaths (7/49) within the follow up period.

Receiver operating characteristic (ROC) analysis was conducted to assess the discriminative performance and the predictive capability of LVD in each region (tumor periphery, tumor centre, and adjacent liver) with tumor recurrence as the end point. Accuracy of a test is measured by the area under the ROC curve. An area under the curve (AUC) of 1 represents a perfect test; an area of 0.5 represents a worthless test. Cut-off points that maximized sensitivity and specificity were established by analyzing ROC curve coordinate points. Using optimal cut-off thresholds determined from ROC analysis, Kaplan-Meier survival curves were generated to compare survival between groups of high and low LVD in the three regions. A logistic regression model was performed using multivariate and univariate analysis.

*P* values of <0.05 were considered statistically significant. All statistical analyses were performed using SPSS (Statistics Package for Social Sciences, SPSS, Chicago, IL).

## 3. Results

### 3.1. Patient Characteristics

A total of 49 patients with histologically proven CLM were included in the study. Median follow-up time for all patients was 27 months (range 4–95 months). Five patients died during the follow-up period. Median time to death was 21 months (range 15–33 months). Patient demographics and clinical characteristics are summarized in Table 1.

All patients had been assessed by a multidisciplinary team consisting of radiologists, HPB surgeons, oncologists, and nuclear physicians prior to commencement of treatment. Standard indications for liver resection of CLM were followed after excluding extrahepatic metastases by multidetector computed tomography (MDCT) of chest and triple phase MDCT of abdomen and pelvis in addition to whole body positron emission tomography with fluorodeoxyglucose integrated with computed tomography (FDG PET/CT) scans. Patients received a combination chemotherapy regimen including oxaliplatin (FOLFOX or capecitabine/oxaliplatin) or irinotecan (FOLFIRI) based regimen.

### 3.2. Spatial Differences in Tumor Lymph Vessel Density

**Figure 1:**
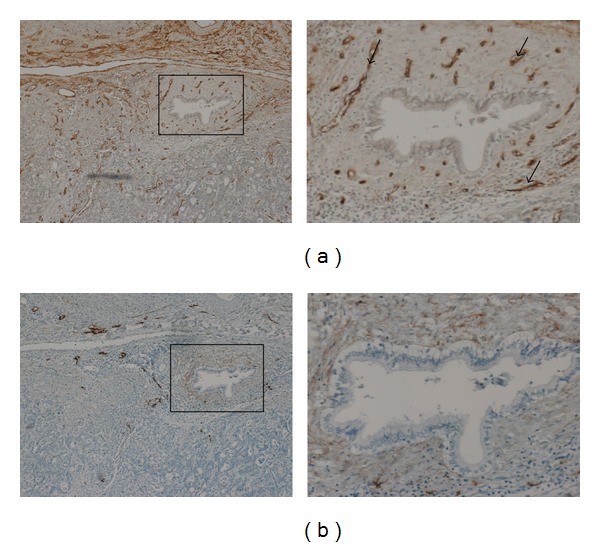
CD34 and D2-40 expression in colorectal liver metastases. (a) Strong staining of blood vessels using CD34, magnified insert. Arrows indicating CD34 positive blood vessels. (b) Lymphatic vessel staining using D2-40; magnified insert indicating the absence of lymphatic vessels in highly vascular region.

CD34 appears to only stain blood vessel endothelial cells, leaving lymphatic endothelial cells negative. Serial immunohistochemistry staining using CD34 and D2-40 antibodies demonstrates the specificity of the markers. No overlapping of vessels was observed between CD34 and D2-40 (Figure 1).

**Figure 2:**
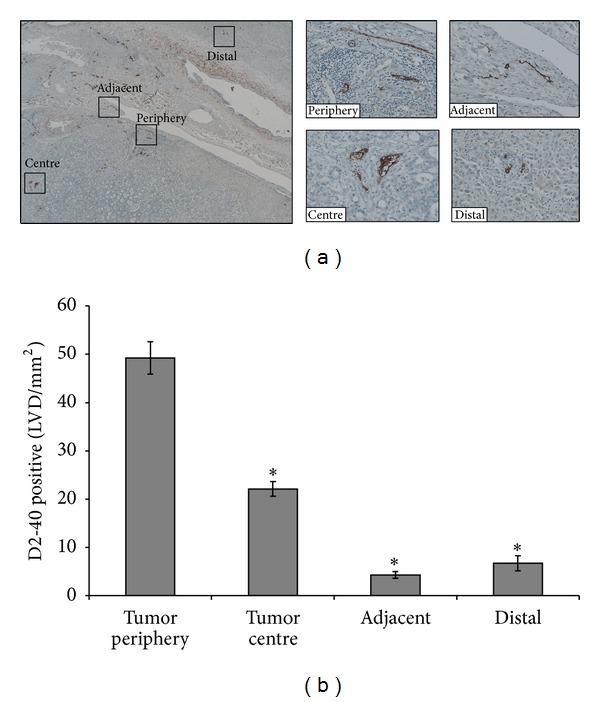
Higher lymphatic vessel density (LVD) in the tumor periphery compared to tumor centre, adjacent, and distal liver. (a) Paraffin embedded section showing lymphatic vessels stained with D2-40 at the tumor periphery and centre, adjacent, and distal liver; magnified inserts of area of interest shown (×400). (b) Enumeration of lymphatic vessel counts revealed higher LVD in the tumor periphery compared to tumor centre, adjacent and distal liver (*P < 0.001).

**Figure 3:**
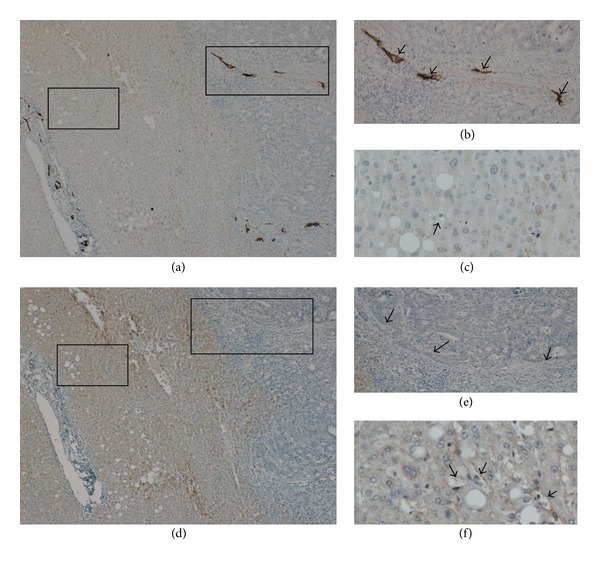
LYVE-1 not able to detect lymphangiogenesis in tumor. (a) Paraffin embedded section showing lymphatic vessels stained with D2-40 (×80). (b) Magnified insert highlighting the strong staining of D2-40 expressing lymphatics within the tumor (arrows) (×400). (c) D2-40 did not stain the liver sinusoids or hepatic blood vessels (arrow) (×400). (d) Serial sections stained using immunohistochemistry with LYVE-1; revealed LYVE-1 was not a specific marker for lymphangiogenesis in the liver (×80). (e) LYVE-1 was not able to detect lymphatic vessels in the tumor periphery where D2-40 was able to detect lymphatic vessels (arrows) (×400). (f) LYVE-1 was expressed in liver sinusoids and hepatic blood vessels (arrows) (×400).

Quantification of D2-40 staining (Figure 2) revealed greater density at the tumor periphery compared to the tumor centre (49.22 ± 24.3 positive LVD/mm² versus 22.1 ± 11.5 positive LVD/mm², *P* < 0.001), adjacent liver (49.22 ± 24.3 positive LVD/mm² versus 4.3 ± 4.9 positive LVD/mm², *P* < 0.001), normal liver (distal to the tumor) (49.22 ± 24.3 positive LVD/mm² versus 6.7 ± 3.1 positive LVD/mm², *P* < 0.001), and benign liver (49.22 ± 24.3 positive LVD/mm² versus 8.7 ± 6.2 positive LVD/mm², *P* < 0.001). LYVE-1, a marker selective for lymphatic vessels, was also carried out. LYVE-1 was found to be expressed in the liver sinusoids but absent from the tumor (Figure 3).

### 3.3. Low Lymphatic Vessel Density Is Associated with Disease-Free Survival Advantage

**Figure 4:**
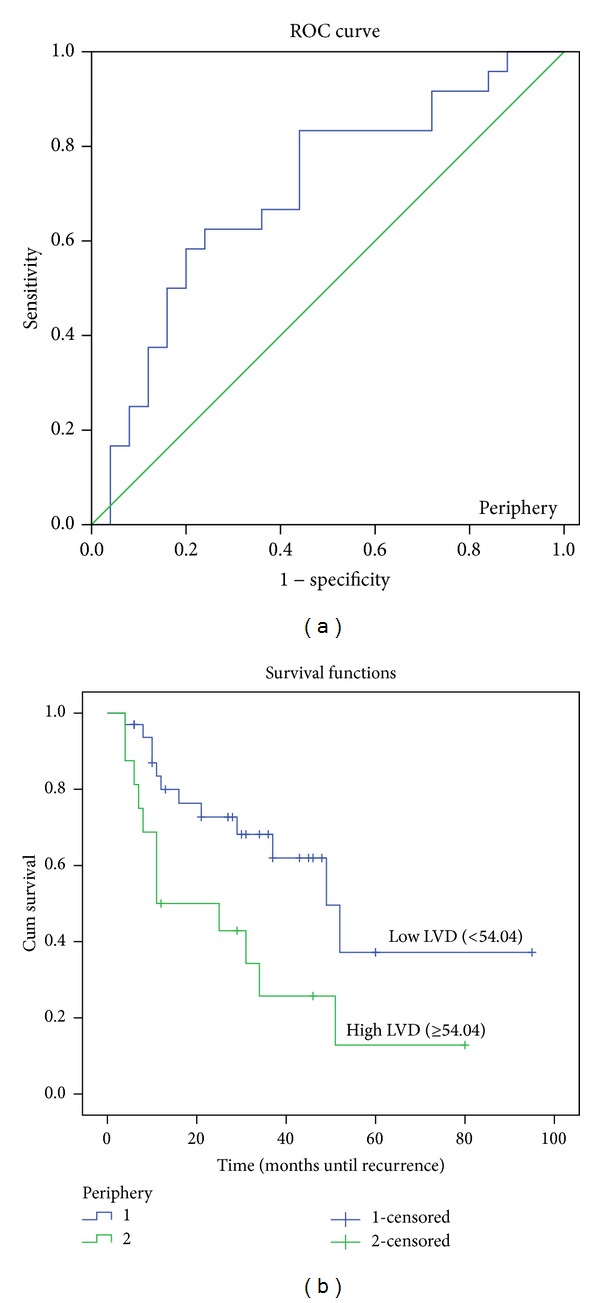
ROC curve and Kaplan-Meier survival curves of lymphatic vessel density (LVD) in the tumor periphery. (a) ROC curve showing the specificity and sensitivity of LVD in the periphery predicting recurrence (AUC = 0.713). (b) High LVD (>54.04) in the tumor periphery correlated with poorer prognosis (*P* < 0.01).

**Figure 5:**
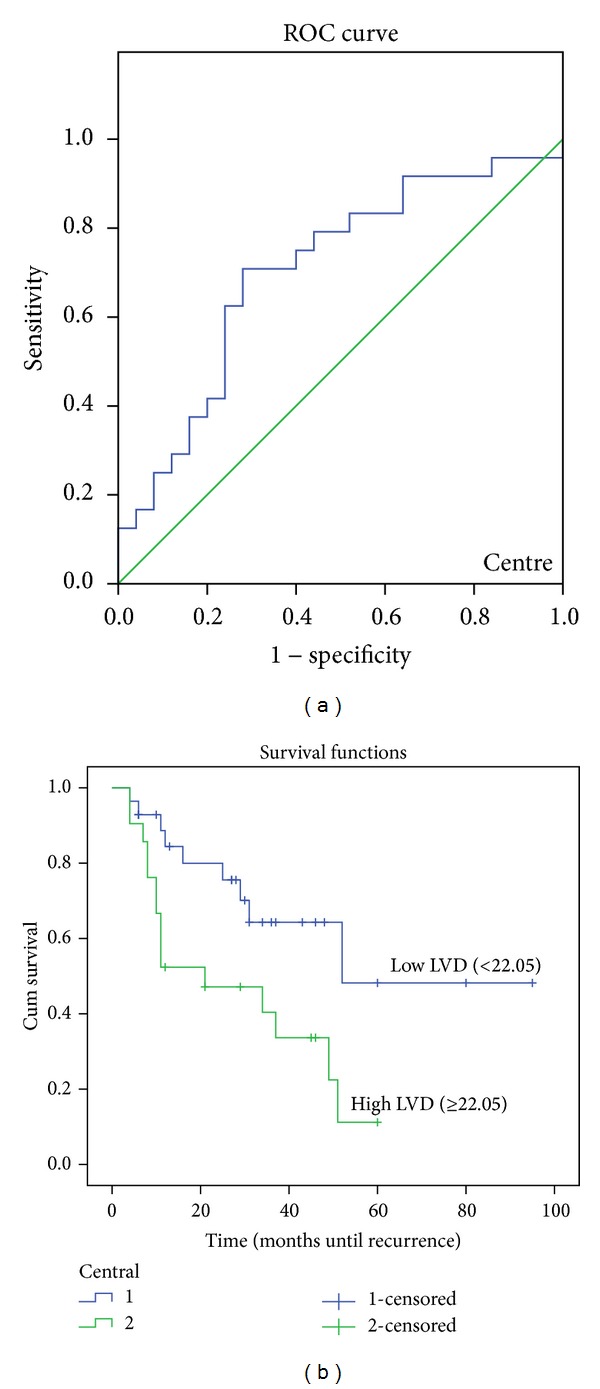
ROC curve and Kaplan-Meier survival curves of lymphatic vessel density (LVD) in the tumor centre. (a) ROC curve showing the specificity and sensitivity of LVD in the centre predicting recurrence (AUC = 0.692). (b) High LVD (>22.02) in the tumor periphery correlated with poorer prognosis (*P* = 0.01).

**Figure 6:**
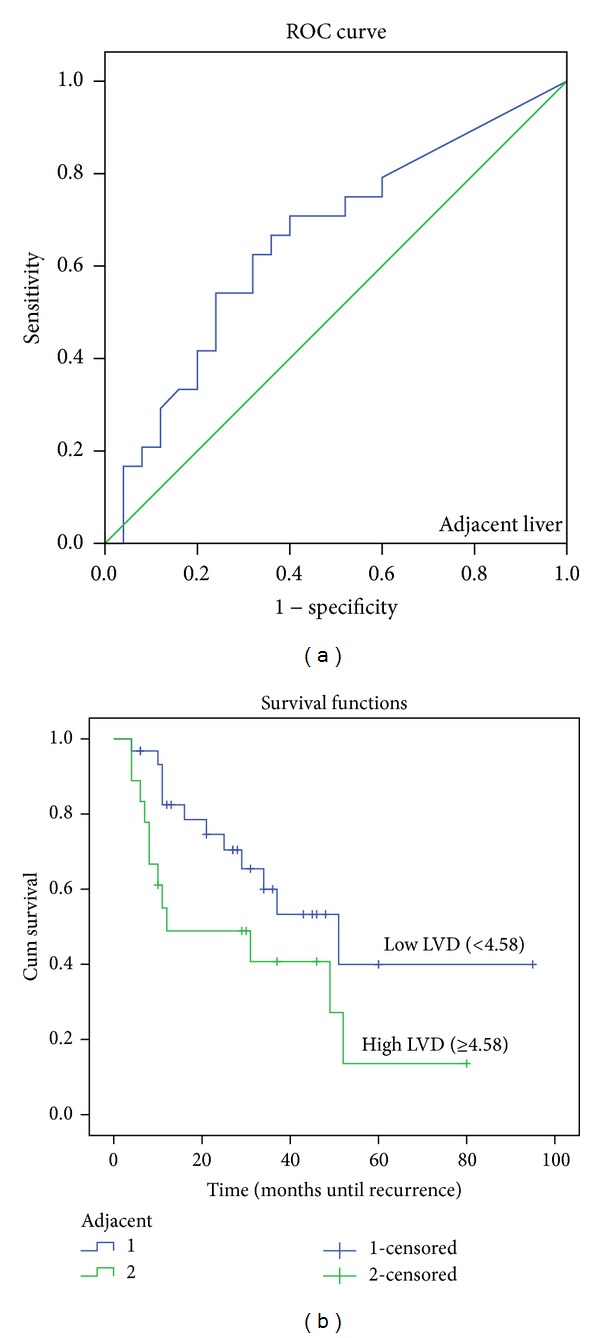
ROC curve and Kaplan-Meier survival curves of lymphatic vessel density (LVD) in the adjacent liver. (a) ROC curve showing the specificity and sensitivity of LVD in the centre predicting recurrence (AUC = 0.708). (b) High LVD (>22.02) in the tumor periphery correlated with poorer prognosis (*P* = 0.01).

The ROC curve in Figures 4(a), 5(a) and 6(a) shows the ability of LVD in the tumor periphery, centre, and adjacent liver to be used as a prognostic marker to predict the likelihood of disease recurrence following hepatic resection. The ROC graph shows a statistically significant ability of peripheral (*P* < 0.01), central (*P* < 0.05), and adjacent liver (*P* = 0.01) LVD to predict disease recurrence. Peripheral LVD was the most discriminative, with an area under the curve (AUC) of 0.713, followed by LVD in the adjacent liver with an AUC equal to 0.708 and central LVD with an AUC equal to 0.692.

According to the optimal cut-off values provided by ROC analysis, patients were categorized into two groups: Low LVD and High LVD in different regions.

A further analysis was performed using Kaplan-Meier disease-free survival graphs. Low D2-40 stained LVD in the tumor periphery (*P* < 0.01), centre (*P* = 0.01), and liver adjacent to the tumor (*P* = 0.018) (Figures 4(b), 5(b) and 6(b), resp.) correlated significantly with disease-free survival.

Table 2 shows that patients, with high LVD in the periphery, centre, and liver adjacent to the tumor, appear to demonstrate close correlation to disease recurrence within the first and second year (*P* < 0.05) after resection. However, only patients with high LVD in the tumor periphery significantly correlated with disease-free survival within the third year (*P* = 0.009) after resection.

In Cox multivariate analysis, taking into consideration the other variables including sex, age, total tumor volume (TTV), largest tumor volume (LTV), and number of lesions, only high LVD in the normal liver adjacent to the tumor showed significant correlation (*P* = 0.046) to disease recurrence following resection (Table 3).

## 4. Discussion

The potential role of lymphangiogenesis in the process of tumor metastasis has been largely overshadowed by the role of angiogenesis [9]. For decades, angiogenesis, the formation of new blood vessels, has been the main focus of research in the pathogenesis of tumor metastases [10]. Over the decades, much knowledge has accumulated linking angiogenesis as an essential step in tumor growth and development [10]. It has been reported that, in the absence of active angiogenesis, the growing tumor may undergo necrosis and apoptosis beyond 1-2 mm² in diameter due to limitation imposed by diffusion [10]. Several studies have focused on the microvessel density (MVD) as a prognostic tool; Hasan et al. observed elevated levels of MVD in CRC to be associated with poor prognosis [11]. Our study is in concordance with those findings and demonstrates significant differences in MVD between different regions of the tumor and adjacent liver. This highlights the spatial differences within the tumor microenvironment and the heterogeneity of the tumor [12].

In contrast to the extensive characterization of molecular mechanisms involved in angiogenesis, research into the role and mechanisms of lymphangiogenesis in cancer has been hampered by singular lack of specific markers [13]. However, recent discovery of lymphangiogenic factor, vascular endothelial growth factor-C (VEGF-C), and specific lymphatic markers (LYVE-1 and D2-40) now allow researchers to identify and focus on the role of lymphangiogenesis in tumor metastases and to determine whether they have prognostic implications.

The recent introduction of LYVE-1 and D2-40 has paved the way for exciting research into the field of lymphangiogenesis: its mechanisms and the possible role these vessels play in the spread and dissemination of tumor cells [7, 13]. Despite the recent focus on lymphangiogenesis research, literature on the role of lymphatic vessels in tumor metastasis has been slow to accumulate with conflicting evidence.

Early studies reported the absence of lymphangiogenesis within the tumor, initially believed to be due to the high interstitial pressure created by rapidly proliferating tumor cells [9]. Due to the absence of intratumoral lymphatic vessels, it was suggested that lymphatic vessels at the periphery were responsible for the spread of tumors [14]. However, more recent studies have reported the presence of intratumoral lymphatics in several different cancers such as breast cancer [15] and colon cancer [16]. Dadras et al. investigated the possibility of using LVD as a marker for prognosis; the study reported high intratumoral LVD in metastatic lesions compared to primary lesions significantly correlated with poor disease-free survival in cutaneous melanoma [17]. In agreement, Saad et al. also reported the presence of intratumoral lymphatics in 46% of cases with stage 1 endocervical adenocarcinoma; however, they observed small and flattened vessels within the tumor, in contrast to the wide open lymphatic vessels found in the tumor periphery, casting a doubt of functionality of intratumoral lymphatic vessels [18]. In contrast to Dadras' findings, Saad et al. showed a significant correlation with peritumoral D2-40 LVD and depth of invasion. Intratumoral LVD was found to have no significant correlation to clinicopathologic parameters [18]. Due to many conflicting results and the absence of a general consensus on the prognostic value of LVD, different types of cancers need to be investigated separately to identify the prognostic value of lymphangiogenesis.

Few studies have investigated LVD as a prognostic marker in CLM and the results have been contradictory [19–21]. Despite recent progress in this field, the potential use of LVD as a prognostic marker in CLM remains unclear. Investigating the expression of VEGF-C, a marker associated with lymphangiogenesis, Matsumoto et al. reported that VEGF-C overexpression significantly correlated with tumor invasion, lymphatic invasion, and lymph node metastases [19]. In contrast, using LYVE-1 as a marker for lymphatic vessels, Brundler et al. observed no significant correlation between LVD and clinical outcome and concluded that LVD had no prognostic value in esophageal adenocarcinoma [20]. One possible explanation for the results reported by Brundler et al. maybe due to the antibody used to identify lymphatic vessels. LYVE-1 does not appear to be a sensitive and reliable marker for lymphatic vessels in all solid tumors. In agreement with previous studies [22], we found that LYVE-1 did not specifically stain lymphatic vessels in the liver. LYVE-1 stained liver sinusoidal endothelial cells and was not able to identify lymphatic vessels in the tumor. LYVE-1 is a known hyaluronan (HA) receptor found on lymphatic endothelial cells; however, the liver sinusoidal endothelial cells play a major role in HA catabolism and as a result also display HA receptors [22]. In addition, Ichida et al. reported elevated HA levels associated with liver injury including cirrhosis and hepatocellular carcinoma (HCC) [23]. It is believed that reduced expression of the scavenging LYVE-1 HA receptors during liver injury and HCC leads to increased HA serum levels [22].

Indeed, in contrast to Brundler et al., Saad et al. reported a significant correlation between LVD and lymph node metastasis and lymphovascular invasion and tumor stage in esophageal adenocarcinoma using D2-40 as a marker for lymphatic vessels [24]. Due to these few and conflicting results, clear consensus on the potential use of LVD as a prognostic marker remains to be elucidated.

Using D2-40 as a specific marker for lymphatic vessels, our data demonstrated that LVD in the tumor periphery and centre and adjacent liver significantly correlated with disease-free survival. LVD in the tumor periphery and centre and adjacent liver correlated with recurrence within the first two years following resection, while LVD in the tumor periphery continued to correlate with disease recurrence three years after resection. However, in the multivariate analysis, only the LVD in the adjacent liver was significantly correlated with disease-free survival. The contradictory results regarding the prognostic significance of LVD in tumor metastases may be due to different patient cohorts, specific tumors included in the analysis, or the method/markers used to detect lymphatic vessels. One of the limitations of this study is that samples were obtained only from a single tumor from each patient. This, therefore, may not necessarily reflect the LVD in other tumors in the same patient and, hence, be a confounding factor.

## 5. Conclusion

This study has demonstrated that D2-40 is effective in identifying lymphatic vessels in human CLM. The monoclonal antibody strongly labeled lymphatic vessels without staining blood vessels as observed in the serial staining of D2-40 and CD34. D2-40 was, therefore, found to be an appropriate and selective marker for lymphatic vessels in CLM. Our results further demonstrate the potential predictive value of LVD detected by D2-40, as a prognostic marker CLM. Despite the limitations imposed by the retrospective nature of the study, relatively short follow-up period, and sampling of single lesions from each patient, the results have established a foundation for investigating what appears to be a potentially significant predictive factor in the long-term survival of patients with CLM. Determining the lymphatic development within tumors may further play a significant role in the selective use of biological agents [25] with the ability to target lymphatics being currently under development.

## Figures and Tables

**Table 1:** Demographic and clinical characteristics.

Variable	Value*
Age (year)	61 (33–84)
Sex (male: female)	29: 20
Number of liver metastases	2 (1–10)
Total volume of tumour (mm^3^)	12845 (502–867600)
Volume of largest tumour (mm^3^)	8181 (381–904778)
Metachronous: synchronous	28: 21

*Data expressed as median (range) or *n* (%).

**Table 2:** Lymphatic vessel density significantly correlates with disease recurrence.

	Tumour periphery	Tumour centre	Adjacent liver
1 year recurrence	0.014	0.007	0.007
2 year recurrence	0.050	0.028	0.034
3 year recurrence	0.009	0.080	0.097

The table summerizes the correlation of LVD in different regions to disease recurrence at 1, 2 and 3 years following resection.

**Table 3:** Cox multivariate regression analysis.

Variables in the Equation								
	*B*	SE	Wald	df	Sig.	Exp (*B*)	95.0% CI for Exp (*B*)	
							Lower	Upper
Age	0.001	0.021	0.002	1	0.964	1.001	0.960	1.044
Sex	−0.521	0.448	1.353	1	0.245	0.594	0.247	1.429
Lesions	0.158	0.252	0.394	1	0.530	1.171	0.715	1.919
Total tumor volume (TTV)	0.000	0.000	0.039	1	0.843	1.000	1.000	1.000
Largest tumor volume (LTV)	0.000	0.000	0.002	1	0.961	1.000	1.000	1.000
Synchronous/metachronous	0.374	0.477	0.616	1	0.433	1.454	0.571	3.700
Tumor periphery	−0.929	0.515	3.252	1	**0.071**	0.395	0.144	1.084
Tumor centre	−0.792	0.544	2.121	1	**0.145**	0.453	0.156	1.315
Adjacent liver	−0.883	0.443	3.982	1	**0.046**	0.413	0.174	0.984

Table demonstrates that when other variables are taken into account, only high LVD in adjacent liver was able to significantly predict disease recurrence.
